# High Mobility Group Box 1 Promotes Aortic Calcification in Chronic Kidney Disease via the Wnt/β-Catenin Pathway

**DOI:** 10.3389/fphys.2018.00665

**Published:** 2018-06-05

**Authors:** Xiucai Jin, Shu Rong, Weijie Yuan, Lijie Gu, Jieshuang Jia, Ling Wang, Honglei Yu, Yifeng Zhuge

**Affiliations:** ^1^Department of Ultrasound, Shanghai Changhai Hospital, Second Military Medical University, Shanghai, China; ^2^Department of Nephrology, Shanghai General Hospital, Shanghai Jiao Tong University School of Medicine, Shanghai, China

**Keywords:** vascular calcification, chronic kidney disease, high-mobility group box1, β-catenin, renal dysfunction

## Abstract

Vascular calcification (VC) is common in chronic kidney disease (CKD), where cardiovascular mortality remains the leading cause of death. Here, we examined the role of high-mobility group box1 (HMGB1), a nuclear DNA-binding protein involved in inflammation, in aortic calcification and renal dysfunction induced by high phosphate in a mouse model of CKD induced by 5/6 nephrectomy. HMGB1 and kidney function markers were measured by ELISA in the serum of CKD patients and in CKD mice. Sections of the aortas of mice were analyzed by immunofluorescence and Alizarin red staining, and protein lysates were generated to analyze the expression of related proteins in response to silencing of HMGB1 or β-catenin by western blotting. Our results showed that serum HMGB1 levels were significantly higher in CKD patients than in healthy controls and related to disease stage. High phosphate promoted the translocation of HMGB1 from the nucleus to the cytosol and aortic calcification in CKD mice *in vivo*, whereas HMGB1 knockdown ameliorated part of renal and vascular function. β-catenin silencing reversed high phosphate-induced calcification and restored renal marker levels. Taken together, our results suggest that HMGB1 is involved in VC associated with CKD via a mechanism involving the β-catenin.

## Introduction

Chronic kidney disease (CKD) is a common multifactorial disorder characterized by kidney damage and associated with an increased incidence of cardiovascular and cerebrovascular disease (Townsend, 2015). Vascular calcification (VC), which is characterized by decreased arterial wall elasticity and blood flow, is associated with kidney disease and is a risk factor for cardiovascular disease and mortality (Demer and Tintut, 2008; Rong et al., 2014). A decline in renal function and proteinuria are associated with cardiovascular diseases, and the incidence of VC and coronary artery calcification (CAC) is high in patients on dialysis (Goodman et al., 2000; Go et al., 2004; Budoff et al., 2013).

The process of VC is similar to that of ossification, as osteogenic proteins accumulate in the arterial wall and promote the differentiation of smooth muscle cells into osteoblasts (Vattikuti and Towler, 2004; Lenhard and Maser, 2017). VC is a multifactorial process that involves genetic factors, molecular pathway interactions, and environmental factors (Pérezhernández et al., 2017). Calcification is mediated by two important pathophysiological pathways, unregulated induction of osteogenesis and loss of mineralization inhibition factors. Osteogenic differentiation is regulated by different pathways and involves oxidative stress, endothelial dysfunction, and proinflammatory factors. The second pathway includes alterations in the pyrophosphate pathway, matrix gla protein, which modulates calcification by inhibiting the interaction between bone morphogenetic proteins (BMPs) and their receptors, OPN and OPG, which inhibit mineralization by acting directly on the vascular wall, and adiponectin, an adipocyte derivative hormone, that modulates osteoblastic differentiation.

VC is also associated with the activation of Wnt/β-catenin signaling (Martínez-Moreno et al., 2012). The canonical Wnt pathway is characterized by the inactivation of a destruction complex targeting β-catenin for proteasomal degradation and the consequent translocation of β-catenin to the nucleus, where it regulates the expression of target genes (Clevers, 2006). The osteoblastic differentiation of vascular smooth muscle cells (VSMCs) is regulated by BMPs and Wnt, and the BMP-Wnt/β-catenin cascade plays a role in VC (Shao et al., 2005; Mikhaylova et al., 2007). High levels of phosphate, which are characteristic of chronic renal failure, are associated with the regulation of arterial calcification mediated by VSMC apoptosis and osteogenic differentiation (Jono et al., 2000; Nishizawa et al., 2005). Renal phosphate excretion is regulated by sodium-dependent phosphate transporters, and their activity is inhibited by FGF23, a bone derived hormone that functions in the regulation of phosphate in the kidney and is associated with atherosclerosis (Russo and Battaglia, 2011). FGF23 activates FGF receptors in a manner dependent on Klotho, a 130 kDA transmembrane protein that binds to FGF23 and functions as an essential cofactor, mediating the regulation of phosphate by FGF23 in the kidney (Kurosu et al., 2006).

Runx2 is an important transcription factor that regulates the differentiation of osteoblasts and chondrocytes and is expressed in atherosclerotic calcified human vascular tissues but not in normal vessels. High phosphate induces the expression of Runx2 in VMSCs through Krüppel-like factor 5, and Runx2 is necessary for high phosphate-induced VC (Zhang et al., 2014). VC is inhibited in Runx2-deficient mice *in vivo*. Runx2 deficiency in mice inhibits the expression of receptor activator of nuclear factor κB ligand concomitant with decreased macrophage infiltration and the formation of osteoclast-like cells in calcified lesions, and Runx2-deficient VSMCs suppress the differentiation of macrophages into osteoclast-like cells, supporting the role of Runx2 in VC (Sun et al., 2012).

High mobility group box 1 (HMGB1) is a nuclear DNA-binding protein that plays an important role in the pathogenesis of kidney diseases mediated by its proinflammatory effects (Chen et al., 2016). HMGB1 initiates innate immune responses by activating cell surface receptors including the receptor for advanced glycation end-products (RAGE) and toll like receptors (TLRs). The new role of RAGE in vascalar osteogenesis has recently been demonstrated, suggesting that RAGE promotes VC by activating Wnt/β-catenin signaling (Menini et al., 2013). Binding of HMGB1 to these receptors activates nuclear factor (NF)-κB, leading to the upregulation of pro-inflammatory cytokines (Zhu et al., 2013). HMGB1 levels are elevated in patients with CKD and associated with glomerular filtration rates and markers of inflammation (Bruchfeld et al., 2008). Atherosclerosis, an inflammatory disease, is accelerated in renal diseases, underscoring the need to understand the link between inflammation, cardiovascular diseases, and CKD.

Here, we used a 5/6 nephrectomized mouse model of CKD fed a high Pi diet to induce aortic calcification and examined the role of HMGB1 in VC associated with CKD.

## Materials and methods

### Patients

A total of 289 CKD patients were recruited from the Nephrology Department at Shanghai General Hospital between January 2014 and June 2016. Sixty-one healthy controls with normal renal function were selected as the control group. The clinical characteristics of healthy volunteers and CKD patients are summarized in Table 1. The present study was approved by the local investigational review board and written informed consent was obtained from all participants.

**Table 1:** Clinical characteristics of healthy volunteers and CKD patients.

	**Healthy controls**	**CKD stage III–IV**	**CKD stage V**	***P*-value**
No. of cases	61	122	167	
Age (years)	58.2 ± 11.7	65.6 ± 10.9	61.3 ± 8.4	0.03
Male gender (%)	52.1	49.2	50.9	0.81
Diabetes mellitus (%)	–	53.3	64.7	
Hypertension (%)	–	56.8	65.1	
Serum calcium (mmol/L)	2.38 ± 0.02	2.42 ± 0.05	2.35 ± 0.03	0.72
Serum creatinine (mg/dL)	0.7 ± 0.2	1.6 ± 0.3	5.4 ± 0.5	<0.001
Blood urea nitrogen (mg/dL)	8.2 ± 0.6	29.5 ± 1.82	38.7 ± 3.15	<0.001
eGFR (mL/min/1.73 m^2^)	94.3 ± 3.2	25.8 ± 4.7	10.4 ± 2.6	<0.001

*Data are shown as a number/frequency or mean ± standard deviation*.

### Samples and analysis

Blood samples were collected in serum separator tubes from patients and healthy participants. Serum is stored in serum storage tubes manufactured by manufacturer BD and the sera obtained were tested within 1 week, centrifuged at 1,000 g for 10 min and serum was stored at −70°C until analyzed. All samples were analyzed simultaneously. Serum HMGB1 levels were measured using a commercially available ELISA kit (Chondrex Inc., Redmond, WA, USA) according to the manufacturer's instructions.

### Induction of CKD in the mouse model

All animal experiments were performed in accordance with the American Animal Protection Legislation. All study protocols were approved by the Institutional Animal Care and Use Committee of Shanghai General Hospital of Shanghai Jiao Tong University School of Medicine. Male C57BL/6J mice aged 8–10 weeks were obtained from SLAC laboratory animal center (Shanghai, China).

According to the experimental design, a total of 112 mice were selected for this experiment. Eight mice were used per group. There were no deaths during feeding, normal drinking, and fast 24 h before surgery. CKD (total of 80 mice) was induced by 5/6 nephrectomy in mice using a standard two stages surgical ablation procedure as described previously (Gong et al., 2016). Briefly, it was performed by the excision of two-thirds of the left kidney, followed by complete nephrectomy of the right kidney 1 week later. Sham-operated control mice (sham) (total 32 mice) were subject to a similar procedure without removal of kidney tissue. After 1 week of recovery, sham and CKD mice (24 of 80 randomly selected) were placed on a normal (0.5%) phosphate diet and CKD mice (the remaining 24) high (1.5%) phosphate diet for 12 weeks. To further explore the role of HMGB1 and β-catenin in CKD *in vivo*, two weeks after 5/6 nephrectomy, Randomly selected 24 and 8 from high Pi and normal Pi mice were administered with 100 μl scramble siRNA (scramble), 16 and 8 from high Pi and normal Pi mice were administered with 100 μl HMGB1 siRNA (siHMGB1), and 8 from high Pi mice were administered with 100 μl β-catenin siRNA (siβ-catenin) lentivirus (HANBIO Biotechnology Co., Ltd, Shanghai, China) at a dose of 10^7^ vector genomes per animal via tail vein injection. The remaining 8 high Pi and 8 normal Pi do not administer. A total of 38 mice failed at each stage of modeling.

All animals were sacrificed on the last day of their feed period. One day prior to sacrifice, mice underwent a 24 h blood collection and serum levels of blood urea nitrogen (BUN), creatinine, calcium, phosphate (Nanjing Jiancheng Bioengineering Institute, Nanjing, China), HMGB1 (Shino-test Co., Kanagawa, Japan) and FGF23 (R&D Systems, Minneapolis, MN, USA) were measured with commercially available kits. Since the ELISA used was specific for both human and mouse samples, mouse HMGB1 can be tested using the ELISA kit mentioned above. For sacrifice, mice were anesthetized with 50 mg/kg pentobarbital, with exsanguination performed via cardiac puncture. The thoracic aorta was harvested from each animal and dissected for Alizarin Red staining, calcium deposition assay and protein extraction.

### Western blot analysis

Tissues from aortas were frozen, homogenized in RIPA buffer on ice, and tissue homogenates were centrifuged at 12,000 g at 4°C for 15 min. The supernatants were stored at −80°C. For separation of nuclear and cytosolic proteins, frozen aortic tissues were homogenized using commercially available nuclear and cytoplasmic extraction kit (Thermo Fisher Scientific Inc., Rockford, IL, USA) according to the manufacturer's protocol and stored at −80°C. Protein concentrations were measured using a BCA kit (Beyotime, Jiangsu, China), and equal amounts of protein were separated by SDS-PAGE in 10% polyacrylamide gels (Invitrogen, Carlsbad, CA). Proteins were transferred to PVDF membranes (Invitrogen), which were incubated overnight at 4°C with antibodies against Runx2, klotho, TLR4, β-catenin (1/200, Santa Cruz Biotechnology, Santa Cruz, CA, USA), HMGB1(1/500), β-actin (1/1,000), or LaminB (1/1,000) (Abcam, Cambridge, MA, USA) followed by incubation with goat polyclonal anti-rabbit IgG HRP-conjugated secondary antibodies at 1/2,000 dilution. Blots were developed on autoradiographic film using the ECL Plus western blotting detection system (Amersham Biosciences, Little Chalfont, England). The gray values were determined using a gel image analysis system (Bio-Rad, Hercules, CA). The bands indicated by the arrow in the Supplemental Figures are used in the manuscript.

### Immunofluorescence analysis

Cryosections (5 mm thick) of the aorta were dried, fixed in cold acetone for 15 min, air-dried, and rinsed twice with PBS. Slides were blocked with 5% normal goat serum for 1 h at room temperature, incubated with antibody against HMGB1 (1/200, Abcam) overnight at room temperature. After three washes in PBS, sections were incubated with rhodamine-conjugated goat anti-rabbit IgG (1/100, Abcam) for 1 h. DAPI solution was added to stain the nuclei. Sections were visualized using a Laser-Scanning Confocal Microscope and imaged (Olympus, Tokyo, Japan).

### Alizarin red staining

Paraffin-embedded aortic sections were deparaffinized with xylene (Sigma, St Louis, MO) and dehydrated in a graded ethanol series and then rehydrated. Sections were washed with PBS three times, dried, and a drop (1 g/L) of Alizarin Red S was added to the surface of sections and incubated for 5–10 min at room temperature. Sections were washed with distilled water, mounted using neutral gum and observed under a microscope (Olympus).

### Aortic calcium deposit content analysis

Aortic sections were desiccated for 4 h at 90°C and weighed. Samples were crushed to a powder with a pestle and mortar and dissolved, using 0.6 N HCl at 37°C for 24 h to decalcify. The calcium content of eluate was measured using the o-cresolphthalein complexone kit (Sigma). Aortic calcium content was normalized to the dry weight of the tissues, based on Wei's methods (Lau et al., 2012).

### Statistical analysis

Data are expressed as mean ± SD. Student's *t*-test was used for comparison between two groups. For more than two groups, mean values were compared using one-way analysis of variance (ANOVA) with comparison between groups by Tukey's *post-hoc* test. A value of *p* < 0.05 was considered statistically significant.

## Results

### High phosphate increases cytosolic HMGB1 levels and induces aortic calcification in a mouse model of CKD *in vivo*

**Figure 1:**
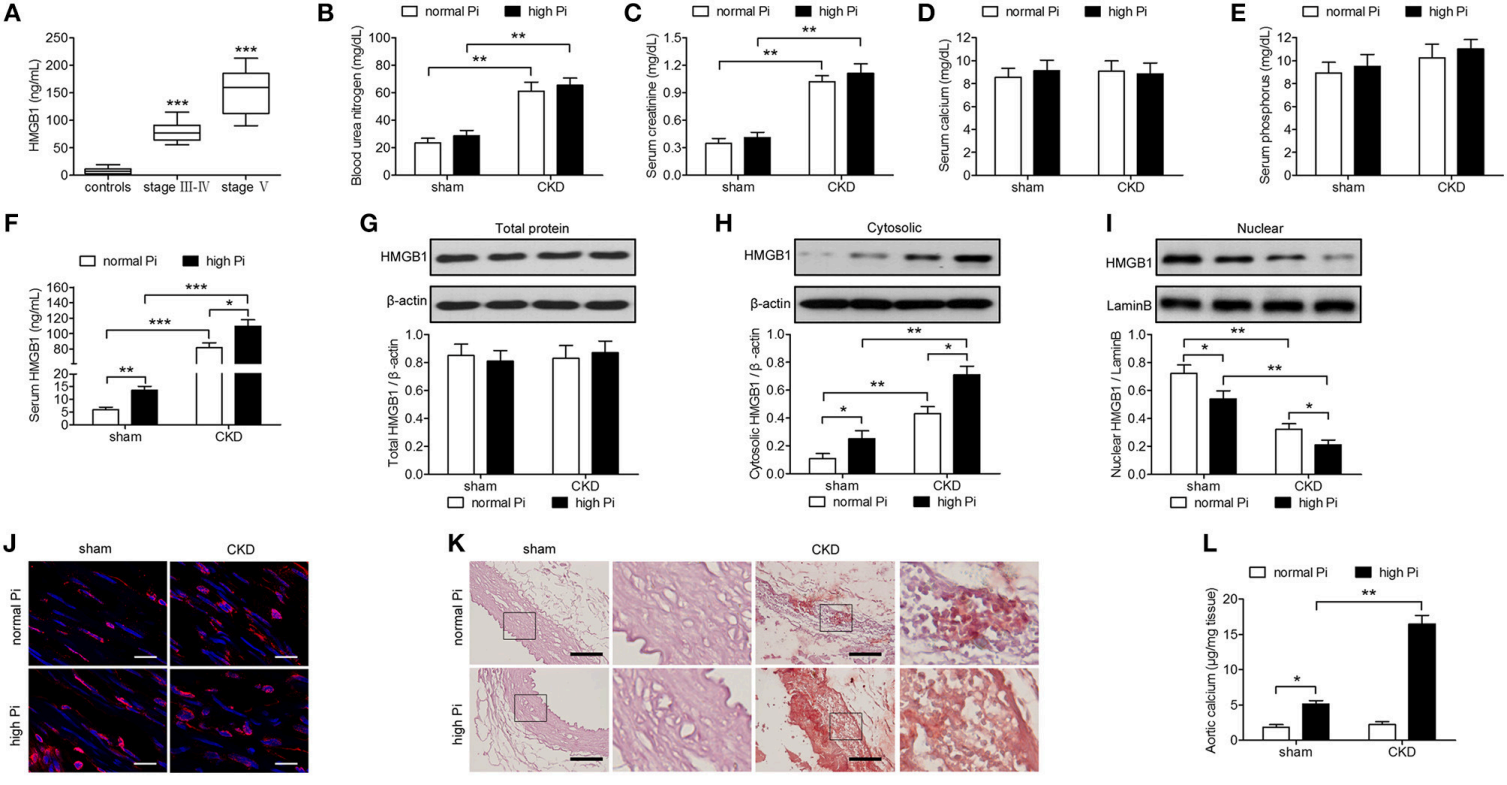
High phosphate increases cytosolic HMGB1 levels and induces aortic calcification in a mouse model of CKD *in vivo*. **(A)** Serum HMGB1 levels of healthy controls and patients with CKD were analyzed by ELISA. ^***^p < 0.001 compared with the controls. **(B–L)** Sham or CKD mice were placed on a normal (0.5%) phosphate diet or high (1.5%) phosphate diet for 12 weeks. Blood urea nitrogen **(B)**, and serum concentrations of creatinine **(C)**, calcium **(D)**, phosphorus **(E)**, and HMGB1 **(F)** were measured by ELISA. Western blot analysis of HMGB1 in total lysates **(G)**, cytosolic **(H)**, and nuclear **(I)** protein extracts of aortas from sham and CKD mice. The western blot image of **(G–I)** in the figure is composite and are obtained from Figures S1–S3. Quantification of HMGB1 expression relative to that of β-actin or LaminB. **(J)** Immunofluorescence detection of HMGB1 [red; nuclei counterstained with DAPI (blue)]. Scale bar = 25 μm. **(K)** Representative micrographs of Alizarin Red stained sections of aortas. Scale bar = 50 μm. **(L)** Calcium content in the thoracic aortas. *n* = 8 per group. Data are presented as the mean ± SD. ^*^p < 0.05, ^**^p < 0.01, ^***^p < 0.001.

To determine the expression of HMGB1 in CKD, blood was collected from 289 CKD patients recruited from the Nephrology Department at Shanghai General Hospital and 61 healthy controls with normal renal function, and HMGB1 levels were measured by ELISA. CKD stage is associated with glomerular filtration (eGFR) and was divided into I-II, III-IV, and V. Stages III–IV in the clinical manifestations and treatment methods were roughly the same, whereas stage V indicated kidney failure. Therefore, stages III-IV were used in joint statistics. The results showed that HMGB1 levels were significantly higher in CKD patients than in healthy controls and correlated with disease stage (Figure 1A). To examine the efficacy of our mouse model of CKD, renal function markers were measured in sham or CKD mice exposed to a high Pi diet. The results showed that BUN and creatinine levels were significantly higher in CKD than in sham operated mice, with no significant differences between high Pi and normal Pi fed mice (Figures 1B,C). Calcium and phosphorous levels did not differ significantly between sham and CKD mice or high and normal Pi fed mice (Figures 1D,E). However, high Pi significantly increased serumHMGB1 levels in both sham and CKD mice, and HMGB1 was approximately seven-fold higher in CKD than in sham operated mice fed a high Pi diet (Figure 1F). Western blot assessment of HMGB1 in lysates from the aortas of CKD and sham-operated mice and densitometric quantification showed no significant differences inHMGB1 expression in total lysates (Figure 1G; Figure S1); however, cytosolic HMGB1 expression was significantly higher in CKD mice than in sham mice and significantly higher in mice fed a high Pi diet than in those fed a normal diet (Figure 1H; Figure S2). Conversely, nuclear HMGB1 was significantly lower in CKD and high Pi than in sham and normal Pi mice (Figure 1I; Figure S3). The cytosolic accumulation of HMGB1 induced by a high Pi diet was also detected using immunofluorescence staining (Figure 1J). Alizarin red staining of sections of the aorta and quantification of calcium levels showed that a high Pi diet induced aortic calcification, with significantly higher calcium content in CKD than in sham-operated high Pi-fed mice, whereas no differences in calcium were detected between sham and CKD mice fed a normal diet (Figures 1K,L).

### Knockdown of HMGB1 attenuates renal dysfunction in CKD mice fed a high Pi diet

**Figure 2:**
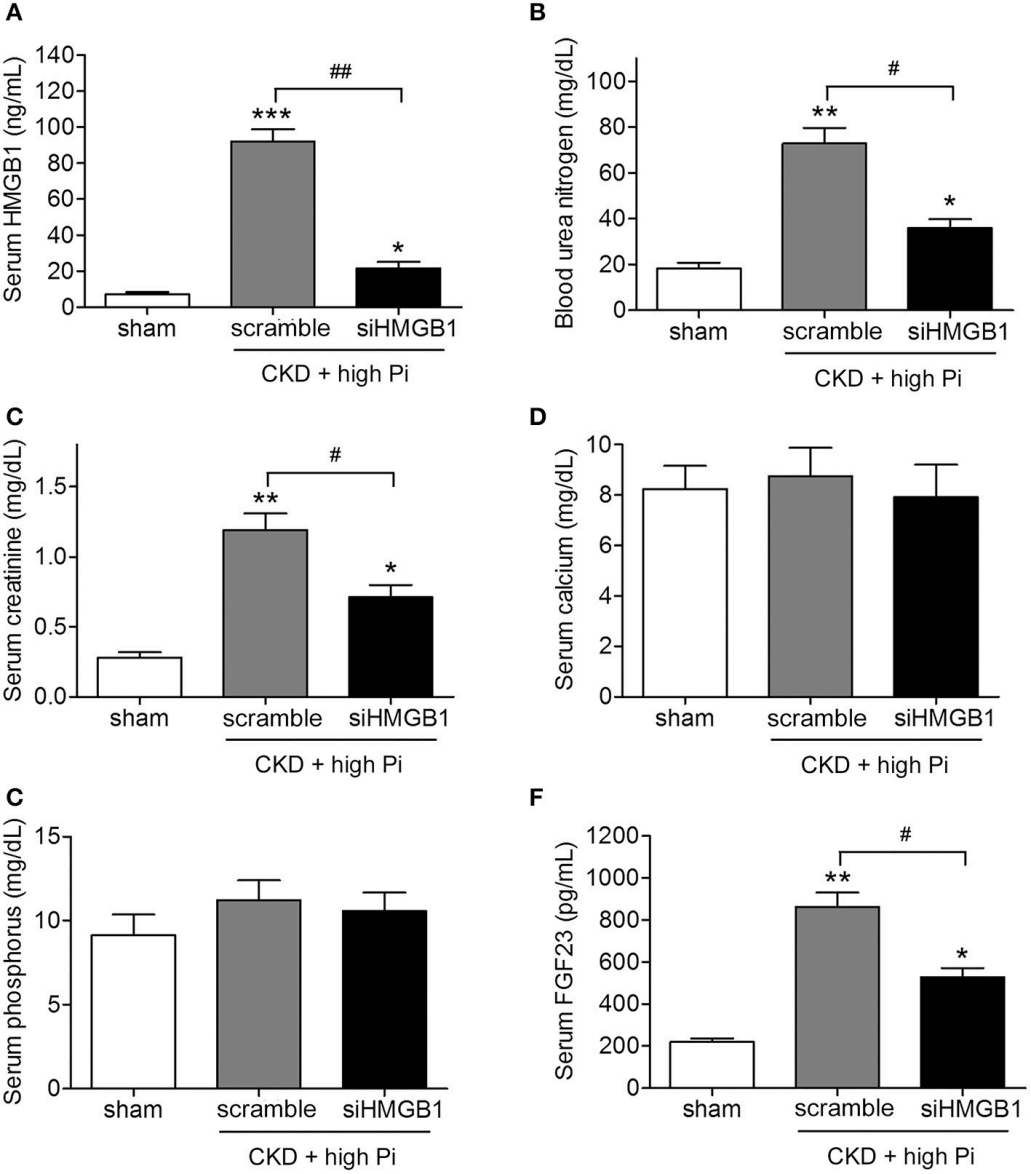
Knockdown of HMGB1 attenuates renal dysfunction in CKD mice fed a high Pi diet. CKD mice were administered with scramble siRNA (scramble) or HMGB1 siRNA (siHMGB1) lentivirus via tail vein injection and placed on high phosphate diet for 12 weeks. Serum concentrations of HMGB1 **(A)**, blood urea nitrogen **(B)**, creatinine **(C)**, calcium **(D)**, phosphorus **(E)**, and FGF23 **(F)** were measured by ELISA. *n* = 8 per group. Data are presented as the mean ± SD. ^*^p < 0.05, ^**^p < 0.01, ^***^p < 0.001 compared with the sham group. ^#^*p* < 0.05, ^##^*p* < 0.01.

To further explore the role of HMGB1 in CKD *in vivo*, mice with induced CKD and fed a high Pi diet were injected with lentivirus expressing siHMGB1 or a scramble control, and serum levels of renal function markers were assessed using specific commercially available kits. Knockdown of HMGB1 led to a significant decrease in serum BUN and creatinine levels without affecting serum calcium or phosphorous levels (Figures 2A–E). Serum levels of FGF23 were approximately 50% lower in siHMGB1 than in scramble control mice (Figure 2F). Taken together, these results indicated that knockdown of HMGB1 ameliorated renal and vascular function, suggesting the involvement of HMGB1 in CKD.

### Knockdown of HMGB1 reduced CKD-induced aortic calcification and inflammation

**Figure 3:**
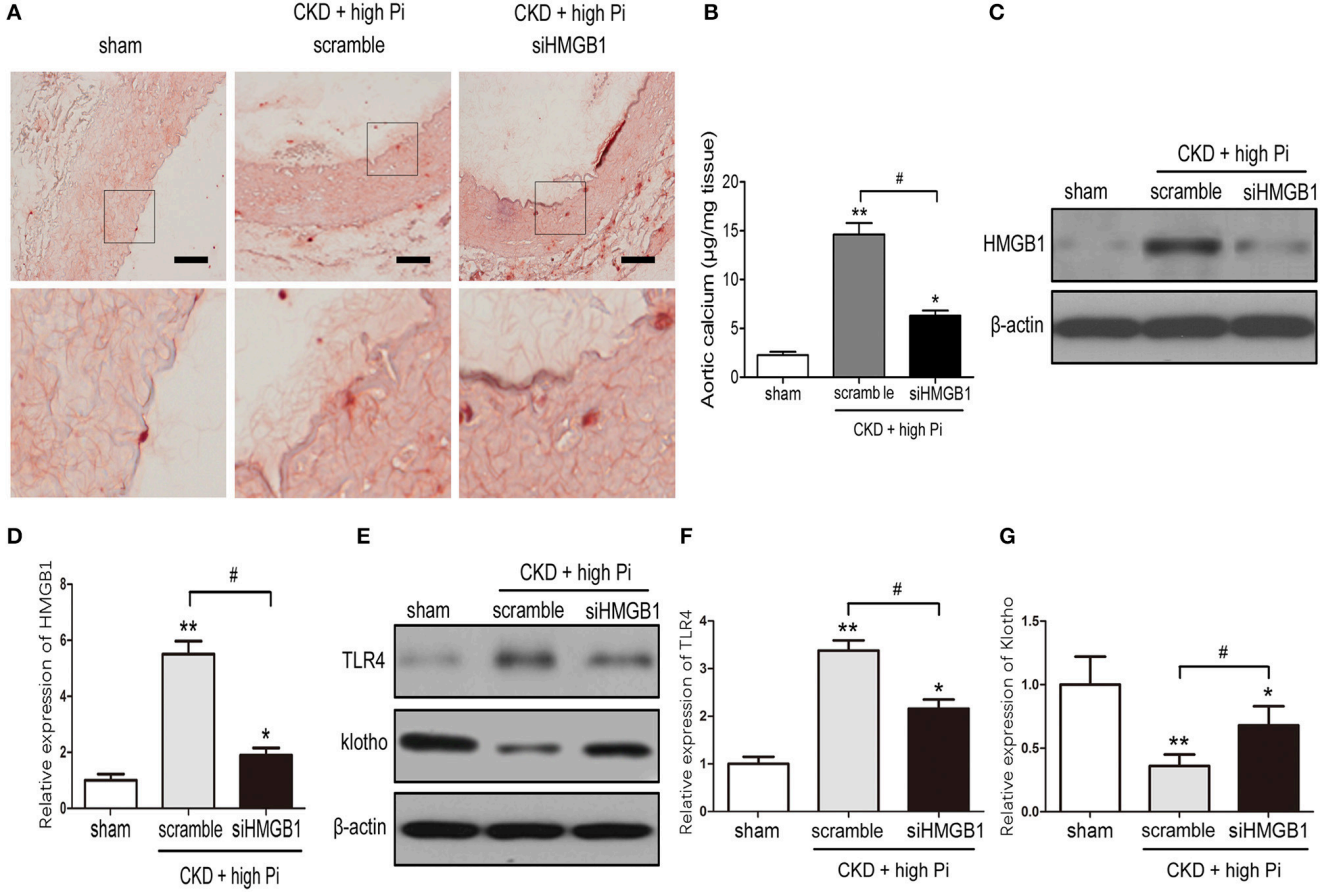
Knockdown of HMGB1 inhibits CKD-induced aortic calcification and inflammation. CKD mice were administered with scramble or siHMGB1 lentivirus via tail vein injection and placed on high phosphate diet for 12 weeks. **(A)** Representative micrographs of Alizarin Red stained sections of the aortas. Scale bar = 50 μm. **(B)** Calcium content in the thoracic aortas. **(C)** Western blot analysis of HMGB1 in the aortas from sham and CKD mice, there are composite and obtained from Figure S4. **(D)** Quantification of HMGB1 expression relative to that of β-actin. *n* = 8 per group. **(E)** Western blot analysis of TLR4 and klotho in the aortas from sham and CKD mice, there are composite and obtained from Figure S5. Quantification of TLR4 **(F)** and klotho **(G)** expression relative to that of β-actin. *n* = 8 per group. Data are presented as the mean ± SD. ^*^p < 0.05, ^**^p < 0.01, compared with sham group. ^#^*p* < 0.05.

The involvement of HMGB1 in VC was further investigated by measuring aortic calcification in CKD mice with HMGB1 knockdown. HMGB1silencing significantly inhibited high Pi induced aortic calcification in CKD mice, as shown by Alizarin red staining and quantification of calcium content in sections of the aorta (Figures 3A,B). In addition, western blotting of lysates of the aorta and densitometric quantification showed that the siHMGB1 lentivirus s knocked down HMGB1 in the aorta, HMGB1 knockdown reversed the high Pi-induced upregulation of TLR4 and downregulation of klotho in CKD mice (Figures 3C–G; Figures S4, S5). Taken together, these results indicated that HMGB1 is involved in VC and the inflammatory responses associated with CKD.

### Involvement of β-catenin signaling in aortic calcification associated with CKD

**Figure 4:**
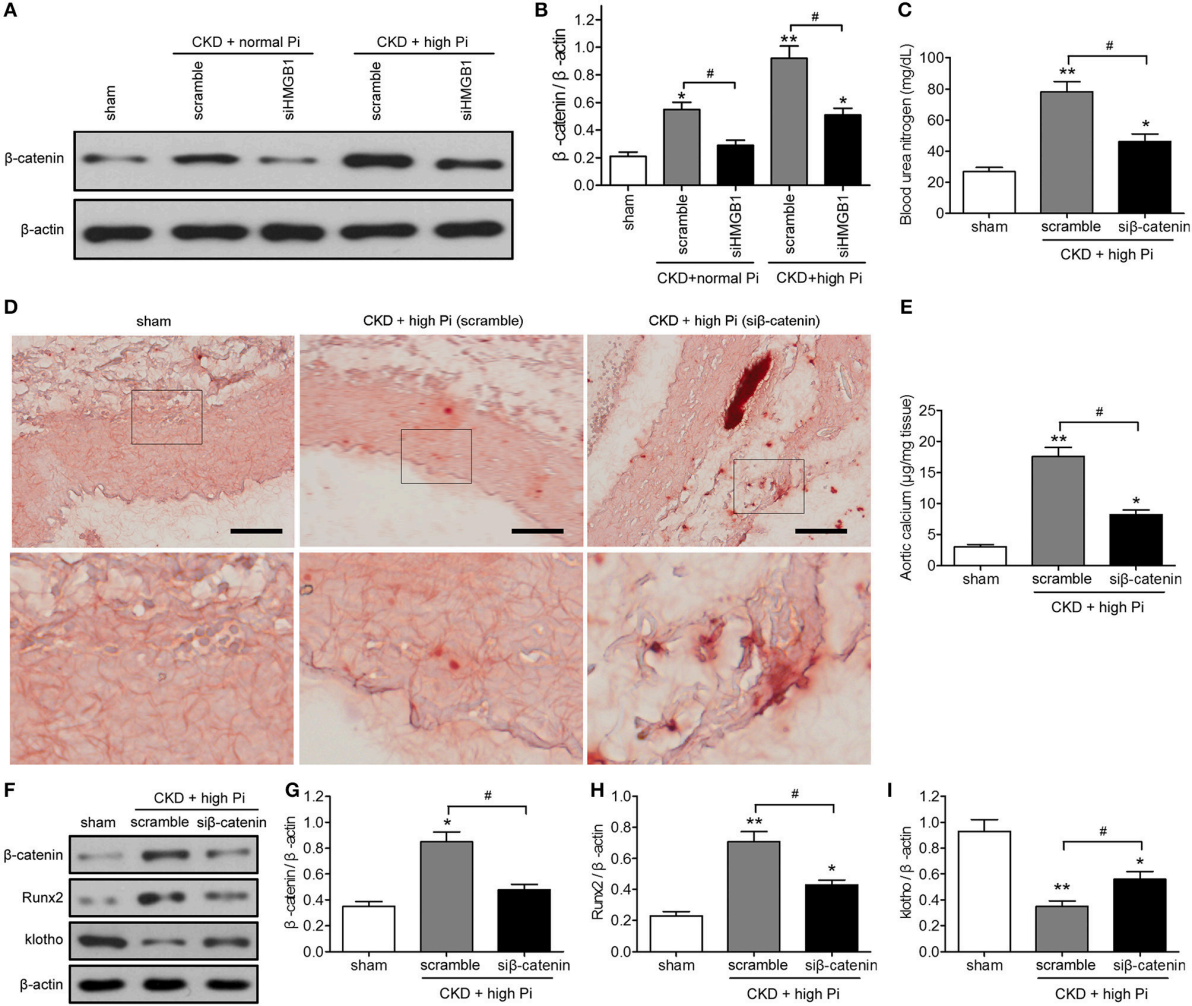
The β-catenin signaling pathway is involved in aortic calcification in a mouse model of CKD *in vivo*. **(A,B)** CKD mice were administered with scramble or siHMGB1 lentivirus via tail vein injection and placed on normal phosphate diet or high phosphate diet for 12 weeks. **(A)** Western blotting assays of β-catenin in aortas from sham and CKD mice, there are composite and obtained from Figure S6. **(B)** Quantification of β-catenin expression relative to that of β-actin. *n* = 8 per group. Data are presented as the mean ± SD. ^*^p < 0.05, ^**^p < 0.01, compared with sham group. ^#^*p* < 0.05. **(C–I)** CKD mice were administered with scramble siRNA (scramble) or β-catenin siRNA (siβ-catenin) lentivirus via tail vein injection and placed on high phosphate diet for 12 weeks. **(C)** Blood urea nitrogen was measured by ELISA. **(D)** Representative micrographs of Alizarin Red stained sections of the aortas. Scale bar = 50 μm. **(E)** Calcium content in the thoracic aortas. **(F)** Western blot analysis of β-catenin, Runx2, and klotho in aortas from sham and CKD mice, there are composite and obtained from Figure S7. Quantification of β-catenin **(G)**, Runx2 **(H)**, and klotho **(I)** expression relative to that of β-actin. *n* = 8 per group. Data are presented as the mean ± SD. ^*^p < 0.05, ^**^p < 0.01, compared with sham group. ^#^*p* < 0.05.

The mechanisms underlying the effects of HMGB1 on aortic calcification in our CKD mouse model were examined by investigating the potential involvement of the β-catenin. Western blot assessment of β-catenin expression showed that HMGB1 knockdown significantly reversed the CKD-induced upregulation of β-cateninin both mice fed a normal and a high Pi diet (Figures 4A,B; Figure S6). Knockdown of β-catenin reversed the CKD/high Pi induced increase in BUN (Figure 4C), indicating that knockdown of β-catenin ameliorated part of renal function. Alizarin red staining and quantification of calcium content in sections of the aorta showed that β-catenin knockdown partially abolished the CKD/high Pi induced calcification of the aorta (Figures 4D,E). Furthermore, silencing of β-catenin reversed the upregulation of Runx2 and downregulation of klotho in the aorta of CKD mice fed a high Pi diet (Figures 4F–I; Figure S7). Taken together, these results indicated that β-catenin signaling is involved in aortic calcification associated with CKD and high phosphate levels.

## Discussion

Cardiovascular disease is the primary cause of morbidity and mortality in CKD, and VC is a risk factor for cardiovascular disease and mortality (Blacher et al., 2001; Valdivielso, 2011). VC is the most important reason for calcium and phosphorus metabolism disorder, when the concentration of calcium and phosphorus in the local and circulating concentrations exceed the phosphate solubility of the integral, insoluble calcium phosphate in the extracellular matrix deposition and lead to VC. Hyperphosphatemia is a risk factor for cardiovascular events in CKD, and a high serum level of phosphorous is a predictor of mortality in CKD (Kestenbaum, 2011; Palmer et al., 2011). In addition, hyperphosphatemia is associated with VC in animal models of CKD, and mice with targeted deletion of FGF23 or klotho show hyperphosphatemia and VC (Stubbs et al., 2007; El-Abbadi et al., 2009; Giachelli, 2009). In the present study, we examined the role of HMGB1 in VC and kidney function in a mouse model of CKD exposed to a high phosphate diet and explored the underlying mechanisms. Our results showed that high Pi induced aortic calcification in parallel with the translocation of HMGB1 from the nucleus to the cytosol, and CKD injury and aortic calcification involved the β-catenin.

HMGB1 levels were measured in a cohort of CKD patients and compared with those in healthy controls. Our results showed that HMGB1 was increased in CKD in correlation with disease stage and the upregulation of markers of kidney dysfunction. Consistently, our mouse model of CKD showed increased serum HMGB1 as well as an increase in cytosolic HMGB1 in the aorta concomitant with aortic calcification. In response to certain stimuli, HMG1 is transported from the nucleus to the cytoplasm, where it modulates processes such as autophagy and apoptosis (Tang et al., 2010, 2011). HMGB1 can also be exocytosed via secretory lysosomes, and once released into the extracellular fluid, HMGB1 mediates inflammatory responses by interacting with cellular receptors and surface molecules (Andersson et al., 2014). The role of HMGB1 in CKD is therefore associated with the regulation of inflammation. HMGB1 is also involved in cardiovascular calcification, which is an inflammatory disease and is associated with CKD, and HMGB1 is considered a cytokine that plays a role in bone remodeling and ectopic calcification (Yang et al., 2008; Chen et al., 2017). Several mechanisms mediating the effect of HMGB1 on promoting VC have been proposed. HMGB1 induces osteogenic differentiation in many cell types and it has been shown to upregulate Runx2 (Meng et al., 2008; Lin et al., 2016). HMGB1 regulates the expression or release of TGF-β and BMP2, which activate osteogenic differentiation and lead to the induction of VC (Gao et al., 2016). HMGB1 mediates inflammatory responses in the arterial wall, and HMGB1-induced inflammation promotes atherosclerotic calcification and VC (Chen et al., 2017). HMGB1 release is regulated by oxidative stress, and HMGB1 in turn promotes ROS formation via a feedback loop (Mohammad et al., 2015; Yu et al., 2015). Since ROS and oxidative stress are involved in the pathogenesis of VC, the relation between HMGB1 and oxidative stress is one of the mechanisms underlying its effect on promoting VC (Al-Aly, 2011). The present results indicate that HMGB1 release is modulated by phosphate levels in an animal model of CKD and involved in aortic calcification.

Angiogenesis has been proposed as an essential mechanism underlying VC, and extensive neovascularization is observed in areas of calcification (Johnson et al., 2006). The association between angiogenesis and calcification may involve a multifactorial process mediated by vascular endothelial cells, SMCs, pericytes, circulating and resident osteoprogenitors, and osteoblastic cells. In addition cytokines such as BMP-2 and−4, and vascular endothelial growth factor stimulate the migration and differentiation of osteoblasts. Other angiogenic factors found in osteoblastic plaque include bone sialoprotein and OPN. Another process associated with VC is apoptosis of VMSCs. BMPs induce apoptosis in pulmonary artery SMCs through the downregulation of the antiapoptotic factor Bcl2 (Zhang et al., 2003). Apoptosis inhibition was shown to suppress calcification, whereas stimulation of apoptosis increases the rate of calcification (Proudfoot et al., 2000).

Knockdown of HMGB1 decreased serum BUN and creatinine levels in parallel with a marked decrease in serum levels of FGF23 in mice. FGF23 levels are increased in CKD, and high FGF23 levels are associated with mortalityand progression to end-stage kidney disease (Fliser et al., 2007). Newly described phosphate and calcium conserving functions of FGF23 may have important implications in CKD, in which FGF23 is elevated. In CKD, decreased kidney function leads to hyperphosphatemia, which is an important risk factor for VC and cardiovascular disease (Erben and Andrukhova, 2017). Therefore, an FGF23-mediated increase in renal-tubular calcium reabsorption may contribute to calcium retention and VC. This is supported by a study reporting a positive association between aortic valve calcification and serum FGF23 levels in patients with CKD (Di Lullo et al., 2015). HMGB1 knockdown also attenuated high Pi-induced inflammatory responses, as demonstrated by the inhibition of TLR4. HMGB1 interacts with TLR2/4 receptors and promotes the translocation of cytoplasmic NF-κB into the nucleus to induce an inflammatory response (Park et al., 2006). In TLR4 deficient (TLR4−/−) mice subjected to ischemia/reperfusion injury, HMGB1 was upregulated in the kidney and serum creatinine and tubular injury decreased in response to treatment with anti-HMGB1 antibodies, and HMGB1 was shown to promote kidney injury through TLR4 (Wu et al., 2010). In another study, preconditioning of wild-type mice with recombinant HMGB1 protected the kidney from TLR4-dependent injury (Wu et al., 2014). These studies support the present results indicating that the effect of HMGB1 on inducing inflammation in the kidney is mediated by TLR-dependent pathways.

The present results suggest the involvement of the β-catenin pathway in aortic calcification and kidney damage associated with HMGB1 release. The involvement of the β-catenin in the osteogenic differentiation and calcification of VSMCs induced by high phosphate or BMP-2 was suggested in our previous study (Rong et al., 2014). Here, we showed that silencing of β-catenin restored part of renal function, decreased aortic calcification, and reversed klotho downregulation in a CKD mouse model fed a high Pi diet. Our results indicate that the effect of HMGB1 release on VC may be mediated by β-catenin activation, providing a potential mechanism underlying the effect of HMGB1 on VC in CKD. HMGB1 was previously shown to activate the Wnt/β-catenin signaling pathway in a rat model of myocardial infarction (Zhou et al., 2012). RAGE, a receptor for HMGB1, promotes aortic smooth muscle cell calcification by activating Wnt/β-catenin signaling (Gao et al., 2017). These studies support the involvement of the association between HMGB1 and β-catenin in the development of VC. Furthermore, high Pi induced aortic calcification via the Wnt/β-catenin pathway in a 5/6 nephrectomized mouse model, and this effect was mediated by the transcriptional regulation of Pit1, a sodium-dependent phosphate cotransporter that plays a role in VSMC calcification (Yao et al., 2015). These findings support the importance of β-catenin signaling in VC associated with high Pi in CKD.

In conclusion, we showed that HMGB1 is upregulated in patients with CKD and in a mouse model of CKD and related to high Pi-induced VC through a mechanism involving the β-catenin. These results provide a potential mechanism underlying VC, an important risk factor for atherosclerosis development in CKD, and suggest potential therapeutic targets and strategies.

## Author contributions

Planned experiments: XJ, SR; performed experiments: XJ, WY, LG, JJ; analyzed data: LG, LW, HY; contributed reagents: SR; wrote the paper: XJ, YZ; deal with all communications with the journal and responsible for the accuracy of all content: SR.

### Conflict of interest statement

The authors declare that the research was conducted in the absence of any commercial or financial relationships that could be construed as a potential conflict of interest. The reviewer LD and handling Editor declared their shared affiliation.
