# Supplementary figures and images for: High Mobility Group Box 1 Promotes Aortic Calcification in Chronic Kidney Disease via the Wnt/β-Catenin Pathway
Source: Front Physiol. 2018 Jun 5;9:665. doi: 10.3389/fphys.2018.00665 (PMC5996195; doi:10.3389/fphys.2018.00665)

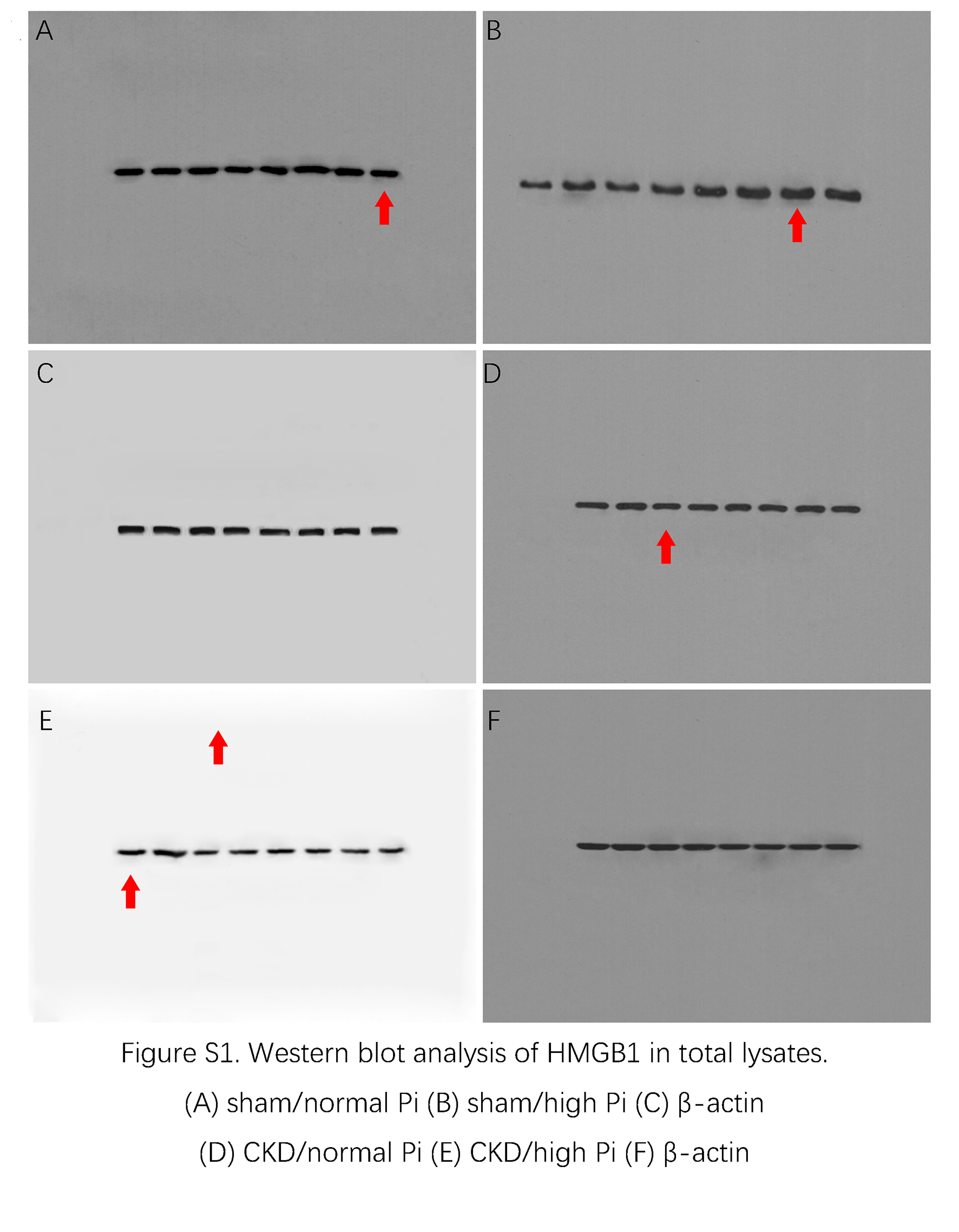

Supplement: Supplementary file 1 [file Image_1.TIF]

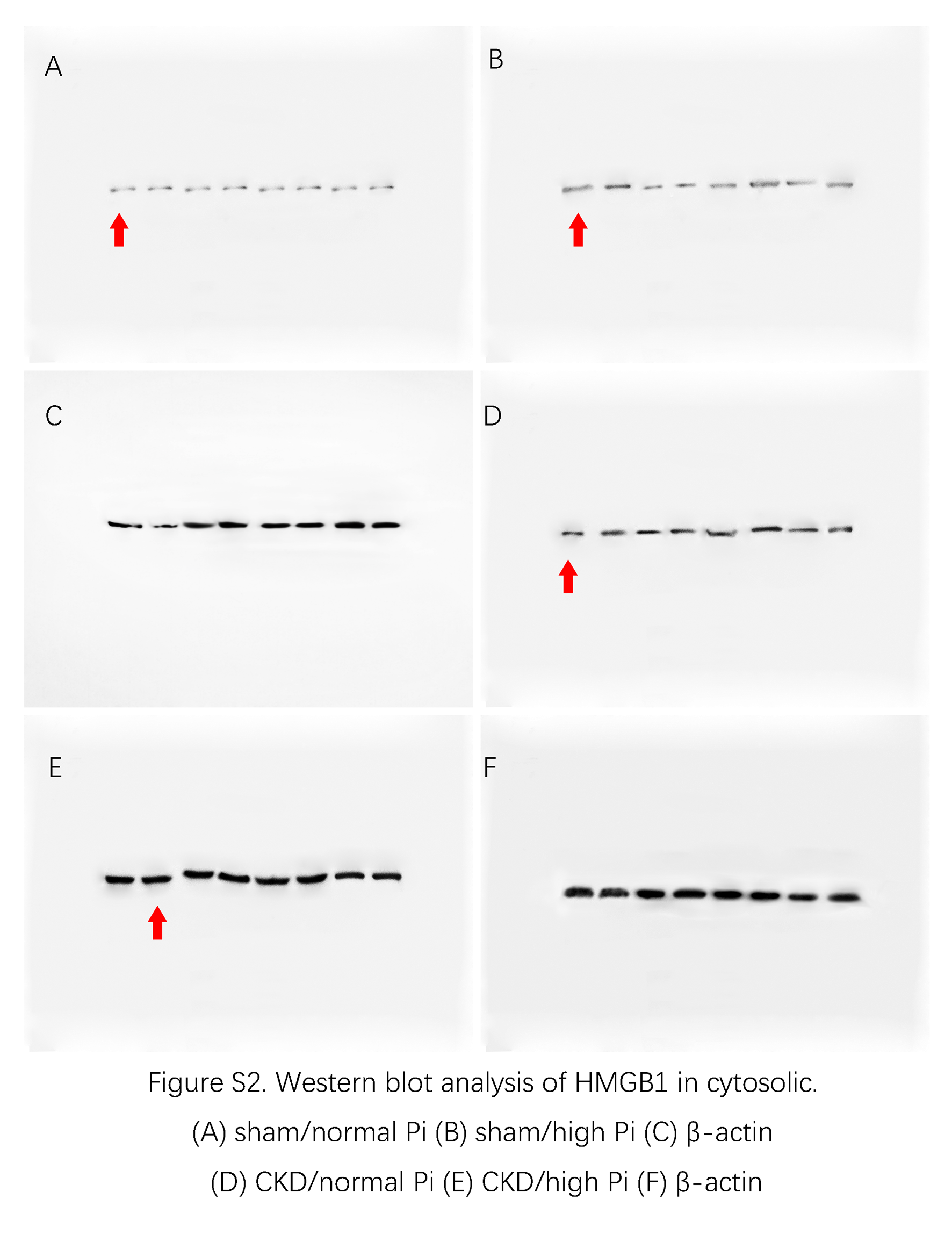

Supplement: Supplementary file 2 [file Image_2.TIF]

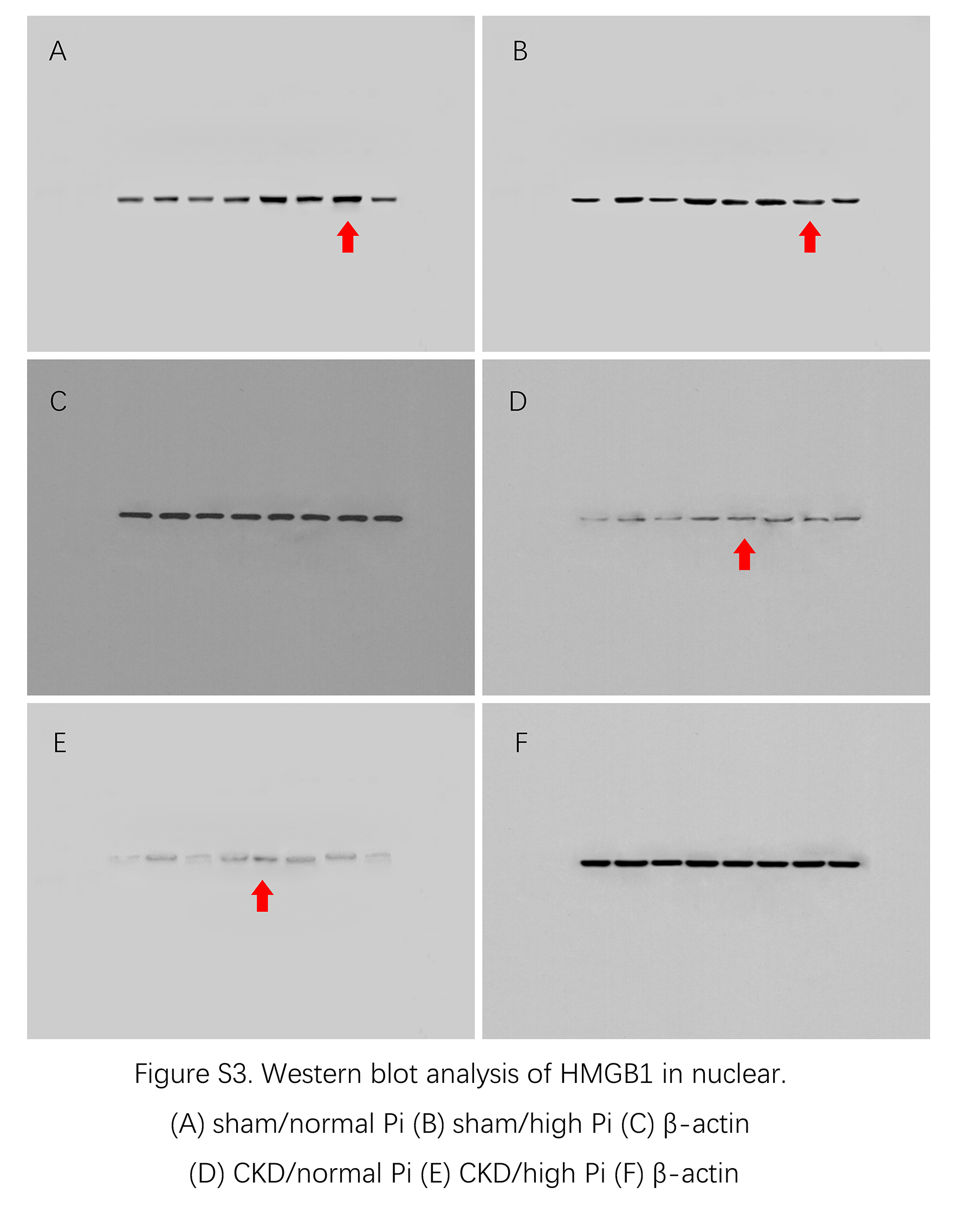

Supplement: Supplementary file 3 [file Image_3.TIF]

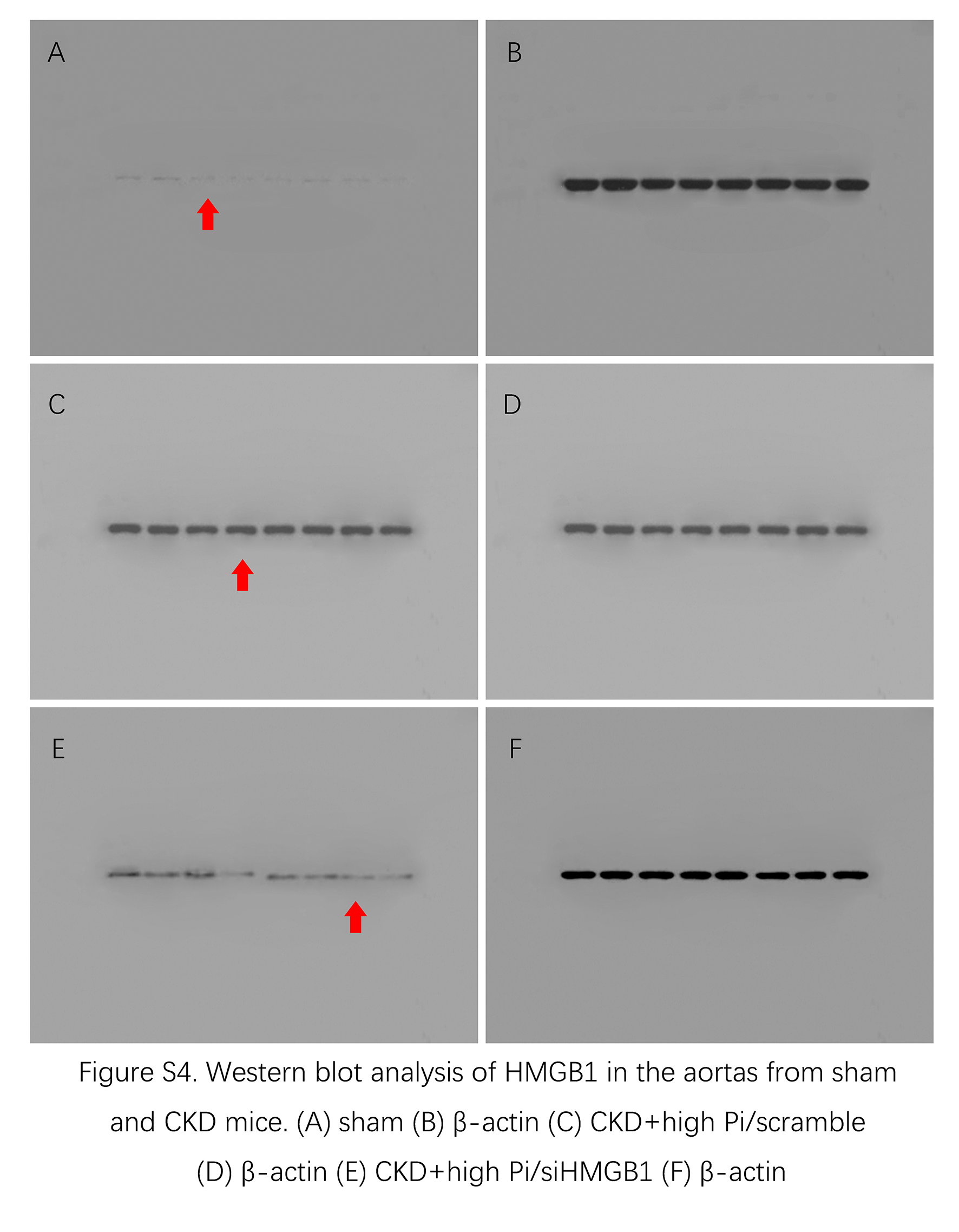

Supplement: Supplementary file 4 [file Image_4.TIF]

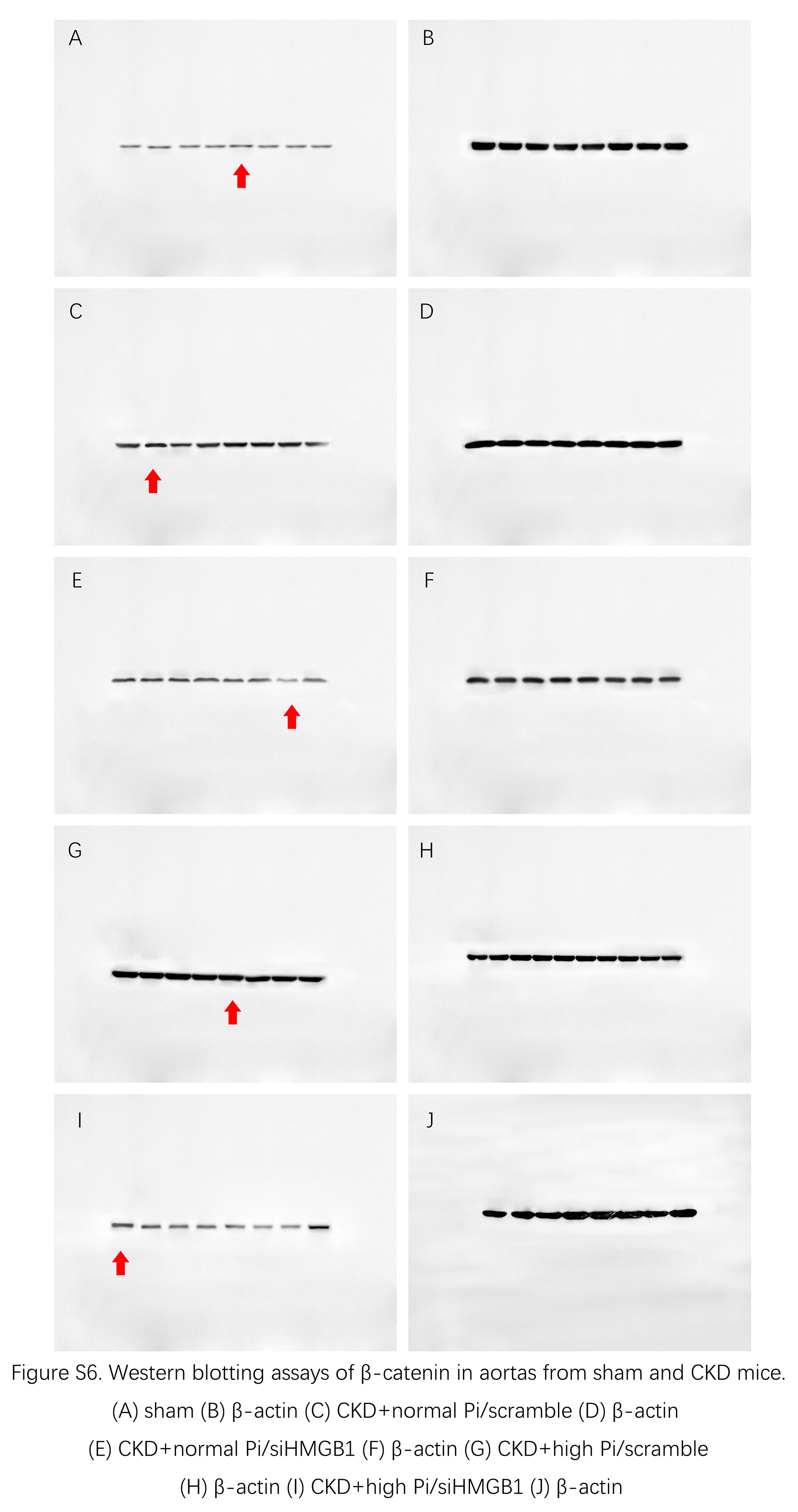

Supplement: Supplementary file 6 [file Image_6.TIF]

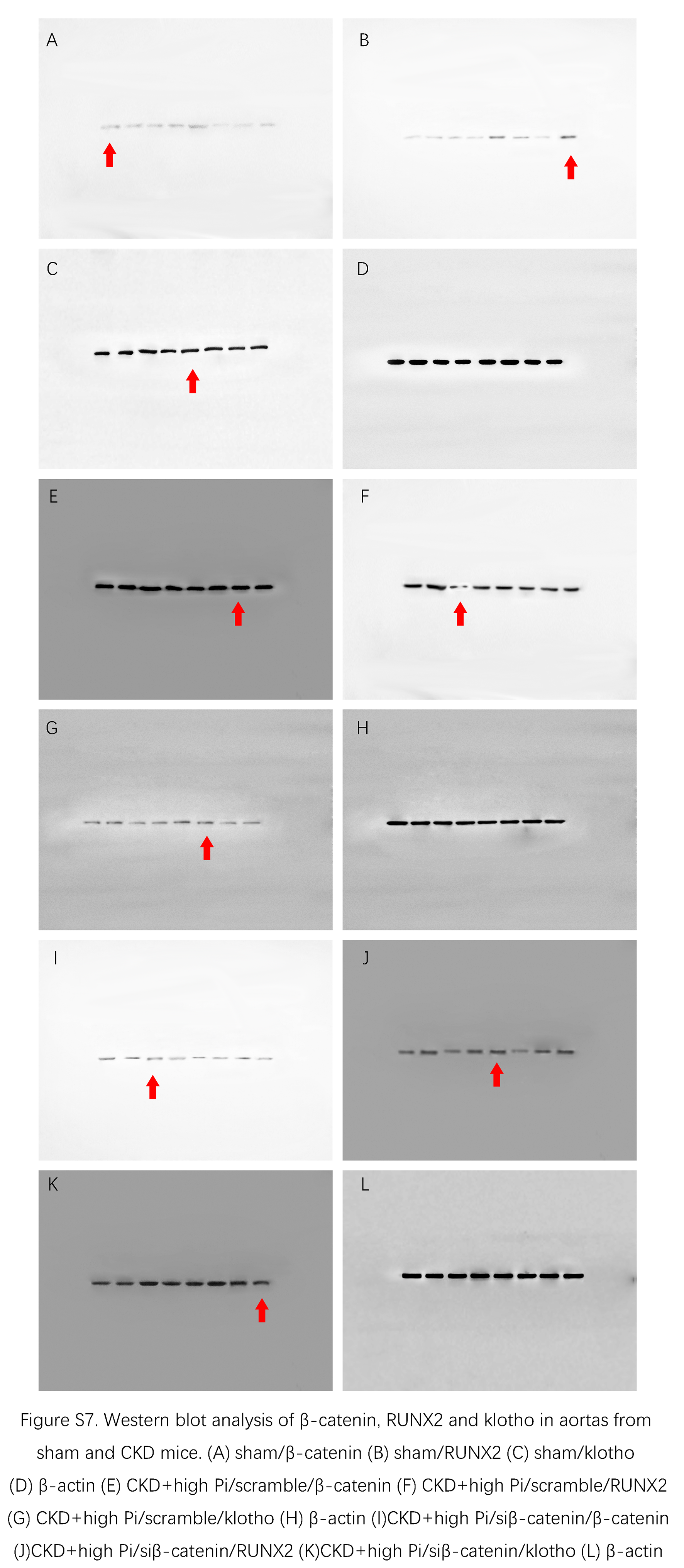

Supplement: Supplementary file 7 [file Image_7.TIF]
