# Supplementary material for: High Mobility Group Box 1 Promotes Aortic Calcification in Chronic Kidney Disease via the Wnt/β-Catenin Pathway
Source: Front Physiol. 2018 Jun 5;9:665. doi: 10.3389/fphys.2018.00665 (PMC5996195; doi:10.3389/fphys.2018.00665)
Supplement: Supplementary file 5 [file Image_5.pdf]

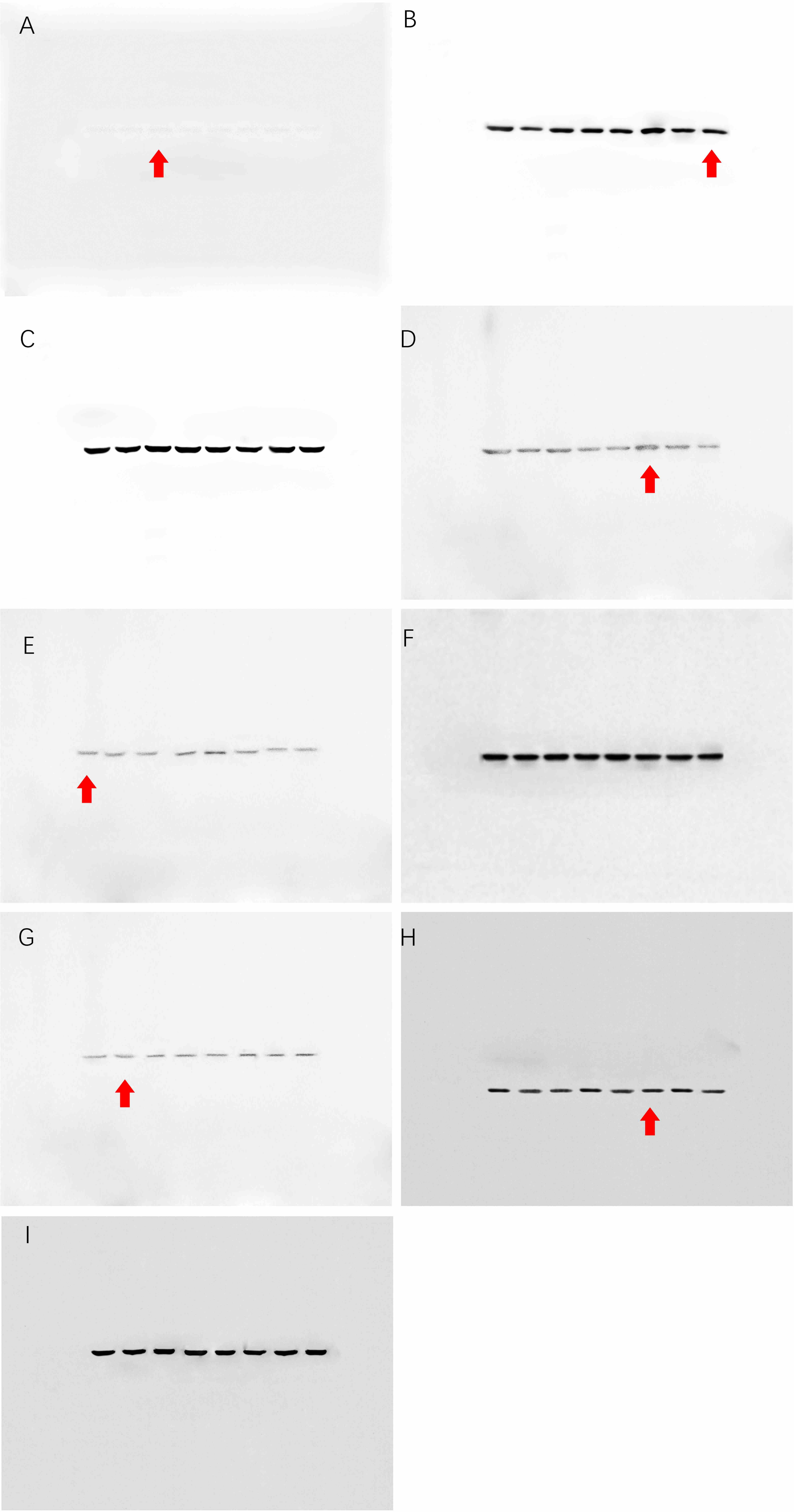

Figure S5. Western blot of TLR4 and klotho in the aortas from sham and CKD mice. (A) sham/TLR4 (B) sham/klotho (C) β-actin (D) CKD+high/scramble/TLR4 (E) CKD+high/scramble/klotho (F) β-actin (G) CKD+high/siHMGB1/TLR4 (H) CKD+high/siHMGB1/klotho (I) β-actin
